# Long‐Acting Injectables: A Strategy to Mitigate Nonadherence in Bipolar Disorder

**DOI:** 10.1111/bdi.70005

**Published:** 2025-02-14

**Authors:** Justin Faden, Elina Maymind

**Affiliations:** ^1^ Department of Psychiatry Lewis Katz School of Medicine at Temple University Philadelphia Pennsylvania USA; ^2^ Rowan‐Virtua School of Osteopathic Medicine Stratford New Jersey USA

Despite our best efforts, partial or nonadherence to treatment is common in bipolar disorder. Varying definitions of nonadherence make a clear prevalence difficult to determine, but a recent nationwide bipolar disorder cohort study identified rates of nonadherence to treatment to be as high as 60%, with a mean prevalence of 40% [1]. The study included > 33,000 individuals with bipolar disorder, and approximately 60% were nonadherent at least once during the monitoring period. This begs the question, why? Nonadherence to pharmacologic treatment is not unique to bipolar disorder, but rates are notoriously high in mental health conditions. Reasons are multifactorial but include the number of comorbidities, young age, co‐occurring substance use disorders, limited primary support system, psychotic symptoms, intensity of manic symptoms, and limited insight, amongst others [1, 2].

The consequence of nonadherence to treatment, especially in early disease bipolar disorder, can be dire. Manic exacerbations have been shown to result in brain damage, functional and cognitive impairment, and worse outcomes [3, 4]. Additionally, potentially due to increased impulsivity, bipolar disorder is strongly associated with increased loss of life due to suicide. The best way to prevent these exacerbations and deleterious outcomes is by maintaining adherence to efficacious treatment, thereby preserving brain function and quality of life.

In a recent article published in bipolar disorders, Vieta and colleagues expound on the landscape of long‐acting injectable (LAI) antipsychotics for the treatment of bipolar disorder and provide expert consensus recommendations [4]. Key findings include moving past the preconceived notion that LAIs can be used only for bipolar disorder patients with severe disease, and utilizing LAIs as early as possible in the bipolar disease course, ideally during the first manic episode [4]. Historically, LAIs have been reserved for patients with chronic nonadherence to treatment and schizophrenia. However, robust evidence supports that LAIs can enhance fidelity to treatment, reduce psychotic and manic exacerbations, and reduce the risk of rehospitalization when compared to oral antipsychotics [4].

Bipolar 1 disorder can be difficult to treat, and individuals will often require multiple medications. However, polypharmacy has also been shown to reduce adherence [1]. LAIs can lower this burden by limiting the number of daily medications, providing consistent medication serum levels, and eliminating the guesswork about treatment adherence status. Using an LAI as the core treatment allows for rational polypharmacy and the utilization of other medications, such as lithium, in a synergistic manner. However, individuals are often not given the option of an LAI due to lack of healthcare provider awareness.

In recent years, there has been a paradigm shift in the availability of LAI antipsychotic medications, with several new formulations having been brought to market and others in advanced clinical development. These new formulations have varying “amenities of care,” allowing the patient and provider to individualize treatment. Options to consider include the following: method of administration (intramuscular versus subcutaneous), injection intervals (ranging from 2 weeks to 6 months), injection sites, number of initiation doses, duration of oral antipsychotic supplementation, needle size, injection volume, prefilled syringe, dose strength, and approved indication, amongst others [5, 6].

Whether a patient will be receptive towards receiving an LAI depends on how the option is communicated, and LAIs should not be considered as a punitive treatment [6]. If considering an antipsychotic medication for bipolar disorder, LAIs should be offered during the initial treatment discussion, normalizing their use in the early management of bipolar disorder. Moreover, discussing LAIs early in the bipolar disease course can reduce stigma and the perception that it should only be used as a “last resort.” As part of shared decision‐making, the initial conversation should discuss potential benefits and drawbacks of LAIs and what the patient's treatment goals are. Moreover, providers should take the time to review the expanding list of available LAI formulations.

Identifying what an individual values most in a medication, ranging from the tolerability profile to dosing frequency, can enhance the collaborative nature of treatment, strengthen the therapeutic alliance, and optimize care. If an LAI antipsychotic is selected, the various amenities of care should be adequately discussed. However, practical limitations and obstacles to LAIs also exist, such as reimbursement barriers and the impact of telehealth from COVID‐19. As several LAIs are new, insurers may be reluctant to authorize coverage, and if an individual is uninsured, identifying applicable patient‐assistance programs will be needed. Additionally, with the rise of telehealth, healthcare providers are often seeing their patients virtually, making administering an LAI logistically challenging. Being aware of community resources, such as pharmacies and clinics able to administer injectables, will be necessary.

Nonadherence to treatment is high in bipolar disorder. LAIs are an underutilized pharmacologic option with evidence supporting their efficacy and role in maintaining fidelity to treatment. As their utilization increases and research grows, their inclusion in treatment guidelines is likely to follow [4].

## Conflicts of Interest

Justin Faden: Grant support — BioXcel Therapeutics. Consultant — Bristol Myers Squibb, Noven. Elina Maymind declares no conflicts of interest.

## Data Availability

The authors have nothing to report.
